# Influence of Anti-TNF and Disease Modifying Antirheumatic Drugs Therapy on Pulmonary Forced Vital Capacity Associated to Ankylosing Spondylitis: A 2-Year Follow-Up Observational Study

**DOI:** 10.1155/2015/980147

**Published:** 2015-05-19

**Authors:** Alberto Daniel Rocha-Muñoz, Aniel Jessica Leticia Brambila-Tapia, María Guadalupe Zavala-Cerna, José Clemente Vásquez-Jiménez, Liliana Faviola De la Cerda-Trujillo, Mónica Vázquez-Del Mercado, Norma Alejandra Rodriguez-Jimenez, Valeria Díaz-Rizo, Viviana Díaz-González, Ernesto German Cardona-Muñoz, Ingrid Patricia Dávalos-Rodríguez, Mario Salazar-Paramo, Jorge Ivan Gamez-Nava, Arnulfo Hernan Nava-Zavala, Laura Gonzalez-Lopez

**Affiliations:** ^1^Universidad de Colima, 28046 Colima, COL, Mexico; ^2^Centro Universitario de Tonala, Universidad de Guadalajara, 48525 Tonala, JAL, Mexico; ^3^Centro Universitario de Ciencias de la Salud (CUCS), Universidad de Guadalajara, 44340 Guadalajara, JAL, Mexico; ^4^Departamento de Inmunología, Facultad de Medicina, Universidad Autonoma de Guadalajara, 44670 Guadalajara, JAL, Mexico; ^5^Centro Universitario de Investigacion Biomedica, Universidad de Colima, 28040 Colima, COL, Mexico; ^6^Coordinación de Investigacion, Division de Cirugia, Hospital Civil de Guadalajara “Dr. Juan I. Menchaca”, 44280 Guadalajara, JAL, Mexico; ^7^Instituto de Investigación en Reumatologia y del Sistema Musculoesqueletico, CUCS, Universidad de Guadalajara, 44340 Guadalajara, JAL, Mexico; ^8^Hospital General Regional No. 110, Instituto Mexicano del Seguro Social (IMSS), 44716 Guadalajara, JAL, Mexico; ^9^Unidad de Investigacion en Epidemiologia, Clinica, Hospital de Especialidades Centro Medico Nacional de Occidente (CMNO), IMSS, 44340 Guadalajara, JAL, Mexico; ^10^Departamento de Fisiologia, CUCS, Universidad de Guadalajara, 44340 Guadalajara, JAL, Mexico; ^11^División de Genetica, Centro de Investigacion Biomedica de Occidente, IMSS, 44340 Guadalajara, JAL, Mexico; ^12^Instituto de Genetica Humana, CUCS, Universidad de Guadalajara, 44340 Guadalajara, JAL, Mexico; ^13^División de Investigacion en Salud, UMAE, CMNO, IMSS, 44340 Guadalajara, JAL, Mexico; ^14^Facultad de Medicina, Decanato de Ciencias de la Salud, Universidad Autonoma de Guadalajara, 44670 Guadalajara, JAL, Mexico; ^15^Servicio de Medicina Interna, Inmunologia y Reumatologia, Hospital General de Occidente, Secretaria de Salud Jalisco, 45170 Zapopan, JAL, Mexico

## Abstract

*Objective*. To evaluate the effect of anti-TNF agents plus synthetic disease modifying antirheumatic drugs (DMARDs) versus DMARDs alone for ankylosing spondylitis (AS) with reduced pulmonary function vital capacity (FVC%). *Methods*. In an observational study, we included AS who had FVC% <80% at baseline. Twenty patients were taking DMARDs and 16 received anti-TNF + DMARDs. Outcome measures: changes in FVC%, BASDAI, BASFI, 6-minute walk test (6MWT), Borg scale after 6MWT, and St. George's Respiratory Questionnaire at 24 months. *Results*. Both DMARDs and anti-TNF + DMARDs groups had similar baseline values in FVC%. Significant improvement was achieved with anti-TNF + DMARDs in FVC%, at 24 months, when compared to DMARDs alone (*P* = 0.04). Similarly, patients in anti-TNF + DMARDs group had greater improvement in BASDAI, BASFI, Borg scale, and 6MWT when compared to DMARDs alone. After 2 years of follow-up, 14/16 (87.5%) in the anti-TNF + DMARDs group achieved the primary outcome: FVC% ≥80%, compared with 11/20 (55%) in the DMARDs group (*P* = 0.04). *Conclusions*. Patients with anti-TNF + DMARDs had a greater improvement in FVC% and cardiopulmonary scales at 24 months compared with DMARDs. This preliminary study supports the fact that anti-TNF agents may offer additional benefits compared to DMARDs in patients with AS who have reduced FVC%.

## 1. Introduction

Patients with ankylosing spondylitis (AS) present a higher proportion of pleuropulmonary and lung functional abnormalities when compared to healthy controls [1]. Mainly, a prevalence of 18–57% in restrictive respiratory pattern on pulmonary function tests (PFT) has been related to spinal and chest wall mobility limitation and in some extent to disease activity indices [2–5], suggesting that disease activity could play a role. Other research groups have suggested the existence of a subjacent inflammatory process in the lung parenchyma of some patients with AS that leads to a reduction of ventilatory function with repercussion in the forced vital capacity. Şenocak et al. [6], for example, observed abnormalities in 85% of their patients with AS in the high resolution computed tomography. Our research group has recently described that around 57% of AS patients had abnormalities in the lung function tests suggesting a restrictive pattern [7]. A systematic review [8] has identified that, in patients with AS, abnormalities in the HRCT can be found in up to 61%. Different findings include radiographic features of nonspecific interstitial abnormalities in 33%, interlobular septal thickening in 30%, ground glass attenuation in 11.2%, and upper lobe fibrosis in 6.9% [8]. Despite these findings, the information available evaluating an association between treatment and improvement in PFT is scarce. So far, only one prospective clinical trial related to PFT has been published, where a significant improvement on PFT was shown after 12 weeks of treatment with etanercept when compared to placebo [9]. With respect to clinical trials, in 2011, Braun et al. demonstrated superiority in clinical efficacy achieved with anti-TNF agents versus synthetic disease modifying antirheumatic drugs (DMARDs) in AS [10].

Until now, the long-term effect of synthetic DMARD and anti-TNF agents on PFT has not been systematically compared in AS patients. Therefore, the objective of the present study was to evaluate, in a two-year observational study, modifications on pulmonary FVC% as well as other cardiopulmonary indices in patients with AS who had reduced FVC% receiving DMARDs or anti-TNF agents + DMARDs.

## 2. Patients and Methods

### 2.1. Study Design

The study is designed as observational prospective cohort with two-year follow-up.

### 2.2. Patients Selection

We enrolled patients attending an outpatient rheumatology clinic, in a secondary-care center (Hospital General Regional No. 110 of the IMSS) in Guadalajara, Jal., Mexico. To be eligible for the study patients met the following criteria:

(a) to be ≥18 years old, (b) to meet the modified New York criteria [11], and (c) to have pulmonary function tests with a reduced FVC% <80% [12, 13].

### 2.3. Methodology to Evaluate Patients

One-hundred and twenty consecutive AS patients were screened with PFT, seeking abnormalities in the FVC%. Spirometry was performed according to the ATS/ERS Task Force considerations [14, 15]. Other parameters evaluated besides the FVC% were forced expiratory volume in one second (FEV_1_) and the FEV_1_/FVC ratio. Observed values were expressed as a percentage of the predicted value compared with individuals of similar sex, age, weight, and height. Restrictive abnormalities were defined with FVC < 80%, FEV_1_/FVC ≥ 70%, and decreased or normal FEV_1_%. A normal PFT was considered when patients had FVC% > 80%, FEV_1_ > 80%, and FEV_1_/FVC > 80%. Furthermore, we arbitrarily classified the reduced FVC% as follows: mild decrement (≥70–79% of predicted value), moderate decrement (51–69% of predicted value), and severe decrement (50% or less of predicted value). We excluded patients with overlapping syndrome (defined as those patients with a suspected or confirmed connective tissue disease associated to AS such as rheumatoid arthritis, systemic lupus erythematosus, or scleroderma), pregnant patients, and pateints with presence of active infections, cardiac failure grade III or IV, chronic obstructive pulmonary disease, asthma, or pulmonary tuberculosis.

### 2.4. Cohort Assembly

The decision to prescribe DMARDs or the addition of anti-TNF agent + DMARDs was left to attending rheumatologist discretion, which was independent of the study. In our hospital except in well-defined cases where synthetic DMARD may have intolerable side effects, all the patients with a diagnosis of AS initiate with NSAIDs combined with a DMARD (usually sulfasalazine or methotrexate) and anti-TNF agents are prescribed only after failure to this combination. Nevertheless, the rheumatologist can be free to prescribe a second combined synthetic DMARD before the initiation of anti-TNF agents in those patients with reasonable suspicion that they may achieve a satisfactory response. Patients with AS and the presence of uveitis received azathioprine. According to the therapeutic schema for treatment, patients that were escalated to receive anti-TNF therapy continued taking the synthetic DMARD, which was originally prescribed. All the patients that received an anti-TNF agent were tested with a PPD and chest X-rays in order to discard latent tuberculosis. If a patient presented a positive PPD test (>5 mm), or suggestive images on X-rays, they were excluded from initiation of anti-TNF therapy and referred to the specialist in infectious diseases.

Two groups were assembled: (a) DMARDs (*n* = 20): patients receiving a DMARD, including one or more of the following drugs: oral methotrexate: 7.5–15 mg/week and oral sulfasalazine 1–1.5 g/day during the entire study period, and (b) patients who escalated to anti-TNF agent + their previous DMARD (anti-TNF + DMARDs) (*n* = 16). Briefly, as described above, this cohort of escalation to anti-TNF agent was constituted by these patients with AS DMARD treatment who fail to achieve improvement with DMARDs alone; therefore, an anti-TNF treatment was added by the rheumatologist, either etanercept administered as a subcutaneous injection 25 mg twice a week; infliximab 3–5 mg/kg given intravenously at zero, two, and four weeks and thereafter every eight weeks; or subcutaneous adalimumab 40 mg every 2 weeks, during the entire study period.

### 2.5. Cointerventions and Dropouts

During the study period all patients received oral nonsteroidal anti-inflammatory drugs (NSAIDs) and in case of being required one or more shot of intramuscular diclofenac were used as adjuvant treatment for spinal or inflammatory joint pain. Additionally, they could receive oral acetaminophen 500 mg to 2 gr daily for pain. Discontinuation or changes in originally assigned therapy were identified and reported.

### 2.6. Baseline Evaluations

PFT including FVC%, FEV, and FEV_1_/FVC were performed as described previously. A structured questionnaire was used to evaluate demographic and clinical variables including disease duration, smoking, and comorbidities. Patients were assessed by the same trained researcher at baseline (time of the initial prescription by attending rheumatologist) and at 6, 12, 18, and 24 months for the following variables: (a) disease activity according to Bath Ankylosing Spondylitis Activity Index (BASDAI) [16] and (b) functioning according to Bath Ankylosing Spondylitis Functional Index (BASFI) [17]. In order to evaluate repercussions secondary to lung affection (deteriorated FVC%), we used the following indices: (a) Saint George Respiratory Questionnaire (SGRQ) [18], a specific health-related quality of life index (HRQOL) for patients with pulmonary disease that consists of a 50-item questionnaire, evaluating 3 domains: symptoms, activity, and disease impact with 10 multiple choice questions and 40 true or false answers, (b) 6-Minute Walk Test (6MWT) [19], used to evaluate one-time cardiopulmonary functional status, and (c) Modified Borg Scale that provides an individual measurement of dyspnea intensity before and after the 6MWT; this test was used to assess the severity of dyspnea [20].

### 2.7. Follow-Up Evaluations

All patients were followed up with the similar strategy. Follow-up took place at 6-month intervals during a period of 2 years. Throughout each visit, the same researchers completed a questionnaire detailing any change in antirheumatic therapy, adverse events associated with the therapy, and evaluated FVC%, FEV_1_, FEV_1_/FVC, BASDAI, BASFI, SGRQ, 6MWT and the Modified Borg Scale.

### 2.8. Primary Outcome Measure

Response was defined as increment in FVC% based on the statistical difference between the evaluations during the follow-up compared with baseline and with the immediate previous measurement. Additionally, improvement in disease activity, functioning indices, and cardiopulmonary scales were also evaluated as secondary outcome measures.

### 2.9. Discontinuation

Reasons for discontinuation were identified.

### 2.10. Statistical Analysis

Due to the nonparametric distribution of the data and/or small sample size, we used medians and ranges in order to describe quantitative variables and for qualitative variables, frequencies, and percentages. Mann-Whitney *U* test was used to compare quantitative variables including medians of FVC% and clinical characteristics between the two groups: (a) DMARDs and (b) anti-TNF + DMARDs. Chi-square (or Fisher exact test when appropriated) were used to compare proportions of qualitative variables between groups of treatment and McNemar test was used to compare differences in intragroup proportions at 2 different time points. For comparison between FVC% at follow-up regarding the baseline values and at 2 different time points, we used Wilcoxon test, and when the comparisons included 3 or more time points we used Friedman test. Statistical significance was considered as *P* ≤ 0.05. All statistical analyses were performed using SPSS, version 10.0.

### 2.11. Ethics

The study was approved by the Institutional Review Board of the Mexican Institute for Social Security (IMSS) of the participating hospital (approval number IMSS R-2009-1301-63); all patients were informed about the study objectives and signed a voluntary consent prior to inclusion. The study was performed following the guidelines of the Declaration of Helsinki.

## 3. Results

**Figure 1:**
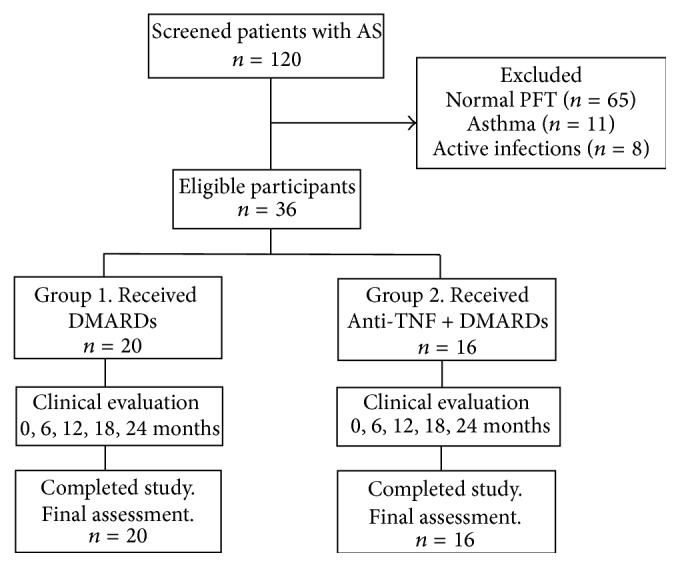
Flowchart of the patients during the cohort. AS, ankylosing spondylitis; LFT, lung function test; DMARDs, disease modifying antirheumatic drugs.

Figure 1 shows the cohort flowchart. We screened 120 patients with AS. Sixty-five patients (54.17%) were excluded because they had normal PFT, 11 (9.17%) with restrictive ventilatory pattern were excluded because they had coexisting asthma, and 8 patients (6.67%) were excluded because they had active infection. Therefore, 36 patients with AS and FVC < 80% were included; from them, 20 were receiving DMARDs and 16 received anti-TNF agents + DMARDs.

There were no significant differences between age, gender, disease activity parameters, lung function test results, cardiopulmonary scales, and SGRQ at baseline (Table 1). A small difference, yet not significant, was observed between DMARDs and anti-TNF + DMARDs groups in disease duration (11 years versus 15 years, resp., *P* = 0.07). In the DMARDs group, 8 patients (40%) were taking methotrexate + sulfasalazine, 5 (25%) sulfasalazine alone, 4 (20%) methotrexate alone, and 2 (10%) methotrexate + azathioprine and 1 patient (5%) received azathioprine alone. In anti-TNF + DMARDs group, 10 patients (62.4%) received etanercept, 5 (31.3%) infliximab, and 1 (6.3%) adalimumab; DMARDs prescribed in combination with anti-TNF were methotrexate alone in 4 patients, azathioprine alone in 2, sulfasalazine in 3, and methotrexate + sulfasalazine in 4 patients, methotrexate + azathioprine in 1, sulfasalazine + azathioprine in 1 and methotrexate + sulfasalazine + azathioprine in 1 patient. All patients continued receiving this medication during the entire study.

Table 2 shows the secondary outcomes observed in both groups. In the intragroup comparisons at 12 months or 24 months versus baseline there was a significant improvement in all secondary variables in the DMARDs group including BASDAI, BASFI, Post-6MWT Borg scale, 6MWT, and total STGRQ%. Similarly in the intragroup comparison between 12 and 24 months versus baseline in the anti-TNF + DMARDs group a significant improvement was observed in all these variables.

### 3.1. Comparison of Changes in FVC%

**Figure 2:**
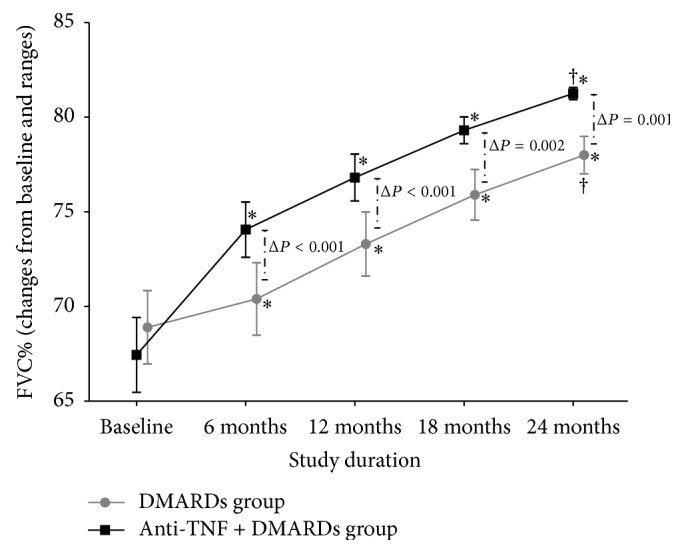
Changes in FVC% during 24 months of the study by treatment group. Modification of FVC (% of predicted value) from baseline to 2 years in the DMARDs (*n* = 20) and anti-TNF + DMARDS group (*n* = 16). Gray circle is DMARDs group, and black square is anti-TNF + DMARDs group. Values are represented as mean and standard error of the mean.  ^*∗*^Comparison in means of each evaluation versus baseline using paired Student *t*-test (*P* < 0.001). ^Δ^Comparison of the absolute change at 6, 12, 18, and 24 months between groups using unpaired Student *t*-test. ^†^Two-factor ANOVA Friedman test *P* < 0.001.

Figure 2 shows the increment observed in FVC% from baseline to the end of the follow-up in the group with anti-TNF + DMARDs versus the group with DMARDs alone. A persistent increment in FVC% was observed in both groups, with this improvement in FVC% being higher in the anti-TNF + DMARDs group and statistical significance being observed for the difference (Δ) of this increment in favor of the anti-TNF + DMARDs since the 6 months (*P* < 0.001) and persisting with significant differences during all the length of follow-up.

### 3.2. Primary Outcomes: Comparison between Groups

Table 3 shows the comparison between groups on the primary outcomes: there was a clear difference in the increase of FVC% in the group receiving anti-TNF + DMARDs at 24 months with respect to DMARDs alone group and there was also a higher increase in FEV_1_/FVC in patients receiving anti-TNF + DMARDs at 24 months with respect to DMARDs alone group (*P* = 0.03). Only 2 patients (12.5%) remained as a restrictive pattern in LFT in the anti-TNF + DMARDs at 24 months compared with 9 (45%) of the patients in the DMARDs alone group. Finally a 87.5% of patients in the anti-TNF + DMARDs group achieved normal FVC% at 24 months (≥80%) compared with only 55% achieving normal FVC% in the DMARDs alone group (*P* = 0.04).

### 3.3. Side Effects

Most frequent adverse events in DMARDs alone group were gastritis and other gastrointestinal side effects (30%), anorexia (25%), and upper airways infections (25%), while in anti-TNF + DMARDs group they were local reactions (44%), upper airways infections (38%), and gastritis and vomiting (13% each). There were no differences in total adverse events between groups of treatment.

## 4. Discussion

In this study we observed that AS patients receiving anti-TNF + DMARDs had a greater significant improvement in FVC% and other pulmonary test parameters at 24 months compared with patients who were taking only DMARDs. To date, the information about the effect of DMARDs in PFT associated to AS is scarce. In our study, the quality of life associated with pulmonary affection (SGRQ) and cardiopulmonary tests including Borg and 6MWT improved significantly in both groups during the 2 years of follow-up.

Our findings are in accordance with the study by Dougados et al. [9] in their 12-week controlled trial, since they found a significant improvement in FVC% and other parameters of lung function tests with etanercept in comparison to placebo. This remarkable study has some differences in comparison with our study: first the study performed by Dougados et al, was a randomised controlled double-blind trial providing high quality information; nevertheless our study has 3 main interesting differences with the previous study. First we had a comparison group with DMARDs and this provides a comparison with drugs that may modify the response in our outcomes; even when compared with DMARDs the addition of anti-TNF agents + DMARDs shows a higher significant improvement in the pulmonary parameters. Second we had a 2 years of follow-up; during the entire study we observed a persistent increment in the FVC% corresponding to the clinical improvement not only in traditional disease activity indices but also in cardiopulmonary variables such as Borg or 6MWT, and finally this cohort represents patients with a wide spectrum of treatments and severity of the disease that is more related to the usual clinical scenario. Nevertheless it is relevant the recognition of the limitations of our exploratory study: the small sample size could reduce the representativeness of the total AS population with a restrictive ventilatory pattern and the accuracy of the observed effects in treatments; likewise, a lack of blindness might induce an expectancy bias. Only patients refractory to DMARDs were included in the anti-TNF + DMARDs group, which could affect the results observed. The use of different combinations of anti-TNF and DMARD therapies diminishes the representativeness of a specific therapy and the results could differ if each specific treatment combination were evaluated separately. Another limitation in this study is the lack of other pulmonary studies such as high resolution computed tomography (HCRT) to evaluate parenchymal damage. Future studies should address whether our observations of a sustained improvement with anti-TNF + DMARDs in FVC% and other cardiopulmonary parameters is correlated with the findings observed in HRCT in these patients.

In conclusion, we found that anti-TNF + DMARDs treatment is superior to DMARDs alone in improving PFT and functional capacity parameters in AS in long-term treatment. Further long-term double-blind, randomized, controlled trials or multicenter cohorts are required in order to corroborate the long-term effects on lung function tests of anti-TNF + DMARDs observed in the present cohort.

## Figures and Tables

**Table 1:** Comparison of selected variables at baseline between treatment groups.

Variable	Treatment groups		*P*
	DMARDs *n* = 20	Anti-TNF + DMARDs *n* = 16	
Males, *n* (%)	14 (70.0)	14 (87.5)	0.26
Age, years	41 (26–67)	45 (27–59)	0.40
*AS characteristics *			
Disease duration, years	11 (1–27)	15 (7–27)	0.07
Past history or present history, *n* (%)			
Enthesopathy	7 (35.0)	11 (68.8)	0.09
Peripheral arthritis	3 (15.0)	6 (37.5)	0.15
Uveitis	3 (15.0)	5 (31.3)	0.42
BASDAI, units	5 (3–8)	5 (4–8)	0.28
BASFI, units	6 (3–7)	5 (3–8)	0.42
Erythrocyte sedimentation rate, mm/h	29.50 (21.0–62.0)	21.0 (12.0–48.0)	0.001
Presence of cervical syndesmophytes, *n* (%)	15 (75.0)	12 (75.0)	1.00
Presence of lumbar syndesmophytes, *n* (%)	12 (60.0)	9 (56.3)	0.20
Cervical or lumbar syndesmophytes bridging, *n* (%)	1 (5.0)	6 (37.5)	0.02
*Cardiopulmonary scales *			
6MWT, mt	282 (235–386)	322 (230–380)	0.89
Pre-6MWT VAS Borg scale	0 (0-1)	0 (0–0.5)	0.35
Post-6MWT VAS Borg scale	2 (0–4.6)	2.2 (0–4.1)	0.37
Development of dyspnea, *n* (%)	9 (45)	7 (44)	0.60
*Health-related quality of life score *			
SGRQ, %			
Symptoms	30 (2–57)	23 (3–43)	0.77
Activity	28 (1–59)	25 (2–57)	0.32
Impact	14 (1–43)	16 (0–45)	0.40
Total	37 (3–58)	29 (3–58)	0.29
*Lung function test *			
FVC (% of predicted)	69 (52–79)	69 (57–77)	0.37
FEV_1_ (% of predicted)	82 (80–90)	82 (81–85)	0.60
Restrictive pattern, *n* (%)	20 (100)	16 (100)	1.00
Severity of restrictive pattern			
Mild (70–79%), *n* (%)	9 (45.0)	8 (50.0)	0.70
Moderate (50–69%), *n* (%)	7 (35.0)	2 (12.5)	
Severe (50% or less), *n* (%)	4 (20.0)	6 (37.5)	
*Treatment *			
Methotrexate, *n* (%)	14 (70.0)	10 (62.5)	1.00
Sulfasalazine, *n* (%)	13 (65.0)	9 (56.3)	0.73
Azathioprine, *n* (%)	3 (15.0)	4 (25)	0.42
Etanercept, *n* (%)	0	10 (62.4)	—
Infliximab, *n* (%)	0	5 (31.3)	—
Adalimumab, *n* (%)	0	1 (6.3)	—
Corticosteroids utilization, *n* (%)	4 (20.0)	2 (12.5)	0.67

AS, ankylosing spondylitis; DMARDs, disease modifying anti-rheumatic drugs; anti-TNF, Tumor necrosis factor-alpha; BASDAI, Bath AS Disease Activity Index; BASFI, Bath AS Functional Index; 6MWT, six-minute walk test; VAS, visual analogue scale; SGRQ, St. George's Respiratory Questionnaire; FVC, forced vital capacity; FEV_1_%, forced expiratory volume in 1 second.

Quantitative variables are presented as median and range; qualitative variables are presented in number (%). Comparisons between proportions were compared with Fisher exact test; comparisons between medians were evaluated with Mann-Whitney *U* test.

**Table 2:** Secondary outcomes. Intragroup changes of selected clinical variables.

Variable	Baseline	12 months	*P *	24 months	*P*
DMARDs group (*n* = 20)					
BASDAI, units^*∗*^	5 (3–8.0)	3 (1–6)	<0.001	1 (0–3)	<0.001
BASFI, units^*∗*^	6 (3–7)	3 (1–5)	<0.001	1 (0–3)	<0.001
Post-6MWT Borg scale^*∗*^	2.2 (0–4.6)	1.0 (0–3.8)	0.001	0.5 (0–2.1)	0.003
6MWT, m^*∗*^	282 (235–386)	308 (280–425)	<0.001	334 (307–440)	<0.001
Total SGRQ%^*∗*^	37 (3–58)	9 (0–53)	<0.001	0 (0–20)	0.003
anti-TNF + DMARDs group (*n* = 16)					
BASDAI, units^*∗*^	5 (4–8)	2 (1–5)	<0.001	0 (0-1)	<0.001
BASFI, units^*∗*^	5 (3–8)	2 (1.0–4.3)	<0.001	1 (0-1)	<0.001
Post-6MWT Borg scale^*∗*^	2.2 (0–4.1)	1.3 (0–3)	0.001	0.5 (0–1.1)	0.002
6MWT, m^*∗*^	322 (230–380)	368 (280–440)	<0.001	400 (315–460)	<0.001
Total SGRQ%^*∗*^	29 (3–58)	7 (0–34)	0.001	0 (0–4)	0.011

DMARDs group, group receiving disease modifying antirheumatic drugs; anti-TNF + DMARDs group, group receiving antitumor necrosis factor agents + DMARDs; BASDAI, Bath Ankylosing Spondylitis Disease Activity Index; BASFI, Bath Ankylosing Spondylitis Functioning Index; 6MWT, six-minute walk test; SGRQ, St. George's Respiratory Questionnaire. Quantitative variables are presented as medians (and ranges); qualitative variables are presented in number (%). *P* values were obtained using Wilcoxon test comparing responses at 12 and 24 months with the baseline. ^*∗*^Significant difference with two-factor ANOVA Friedman test *P* < 0.001.

**Table 3:** Primary outcomes. Comparison in absolute changes on pulmonary function tests between baseline and 24 months in DMARDs group versus anti-TNF+DMARDs group.

Variable	DMARDs *n* = 20			Anti-TNF + DMARDs *n* = 16			Comparison between groups at 24 months (Δ absolute change)
	Baseline	24 months	Absolute change	Baseline	24 months	Absolute change	*P*
FVC %, median (ranges)	69 (52–79)	80 (70–82)	11 (2–18)	69 (57–77)	82 (79–83)	13 (5–23)	0.04
FEV_1_%, median (ranges)	82 (80–90)	86 (82–95)	3 (−2–8)	82 (81–85)	85 (81–90)	3 (−4–6)	0.60
Ratio FEV_1_/FVC, median (ranges)	94.5 (87–112)	97 (90–107)	2 (−15-14)	84 (82–99)	90 (86–99)	4 (0–13)	0.03

Patients with improvement	% achieving improvement					% achieving improvement	Comparison of % achieving improvement

Restrictive pattern, *n* (%)	20 (100)	9 (45)	11 (55.0)	16 (100)	2 (12.5)	14 (87.5)	0.04
Changes of cut-off point in FVC%							
Normal (≥80%), *n* (%)	0	11 (55.0)	—	0	14 (87.5)	—	<0.001
FVC% 70–79%, *n* (%)	9 (45.0)	7 (35.0)	—	8 (50.0)	2 (12.5)	—	
FVC% 50–69%, *n* (%)	7 (35.0)	2 (10.0)	—	2 (12.5)	0	—	
FVC% <50%, *n* (%)	4 (20.0)	0	—	6 (37.5)	0	—	

DMARDs, disease modifying antirheumatic drugs; anti-TNF + DMARDs, antitumor necrosis factor + DMARDs; FVC%, forced vital capacity; FEV_1_%, forced expiratory volume in 1 second. Quantitative variables are shown as median and ranges; qualitative variables are shown in frequencies (%). Absolute change is obtained from the differences at 24 months versus baseline. *P* values for Δ absolute change: comparison between groups of differences in absolute changes at baseline and 24 months. Comparison of the absolute change between groups at 24 month was performed with Mann-Whitney *U* test. Fisher exact test was used to compare the proportion of patients who achieve changes of the cut-off point in FVC%.
